# Scene content is predominantly conveyed by high spatial frequencies in scene-selective visual cortex

**DOI:** 10.1371/journal.pone.0189828

**Published:** 2017-12-22

**Authors:** Daniel Berman, Julie D. Golomb, Dirk B. Walther

**Affiliations:** 1 Department of Psychology, The Ohio State University, Columbus, Ohio, United States of America; 2 Department of Psychology, University of Toronto, Toronto, Ontario, Canada; University of Muenster, GERMANY

## Abstract

In complex real-world scenes, image content is conveyed by a large collection of intertwined visual features. The visual system disentangles these features in order to extract information about image content. Here, we investigate the role of one integral component: the content of spatial frequencies in an image. Specifically, we measure the amount of image content carried by low versus high spatial frequencies for the representation of real-world scenes in scene-selective regions of human visual cortex. To this end, we attempted to decode scene categories from the brain activity patterns of participants viewing scene images that contained the full spatial frequency spectrum, only low spatial frequencies, or only high spatial frequencies, all carefully controlled for contrast and luminance. Contrary to the findings from numerous behavioral studies and computational models that have highlighted how low spatial frequencies preferentially encode image content, decoding of scene categories from the scene-selective brain regions, including the parahippocampal place area (PPA), was significantly more accurate for high than low spatial frequency images. In fact, decoding accuracy was just as high for high spatial frequency images as for images containing the full spatial frequency spectrum in scene-selective areas PPA, RSC, OPA and object selective area LOC. We also found an interesting dissociation between the posterior and anterior subdivisions of PPA: categories were decodable from both high and low spatial frequency scenes in posterior PPA but only from high spatial frequency scenes in anterior PPA; and spatial frequency was explicitly decodable from posterior but not anterior PPA. Our results are consistent with recent findings that line drawings, which consist almost entirely of high spatial frequencies, elicit a neural representation of scene categories that is equivalent to that of full-spectrum color photographs. Collectively, these findings demonstrate the importance of high spatial frequencies for conveying the content of complex real-world scenes.

## Introduction

Everything that our visual system processes begins as collections of low-level features, such as contrasts, colors, orientations, and spatial frequencies. Collectively, these features represent all of the visual information contained in the image. However, semantically meaningful, higher-level concepts, such as the category, identity or affordance of a scene are not directly accessible at the early stages of visual processing. The image information must undergo further neural computations in order to make such semantically complex aspects of the stimuli explicit. As such, one of the primary goals of vision research is to elucidate these computational mechanisms by which the brain utilizes low-level image features in order to extract high-level meaning. Behavioral, neuroimaging and computational modeling studies have made great progress illuminating the roles of color [1, 2], spatial frequency spectra [3, 4], and contours and their junctions [5, 6] with regard to natural scene perception.

In the last few years, several studies have sought to expound upon the neural representations of high-level visual concepts in scene-selective brain regions by reducing them to low-level features [7–10]. For instance, Watson et al. (2014) [9] showed that representations of scene categories in the parahippocampal place area (PPA) can be reduced to a set of oriented filter banks, concluding that there is no true categorical representation in that region. Most recently, Watson et al. (2016) [10] reported that the neural activity of a large visual ROI that included scene-selective regions could discriminate between high and low spatial frequencies in an image better than between natural and man-made image content. When restricting the analysis to only the PPA, they found the opposite pattern of results.

Here, we take a different theoretical approach to probing the role of low-level features in visual perception. Rather than juxtaposing representations of low-level visual features with high-level content, we probe the visual system by asking *how much image content* is carried by different low-level features. Specifically, we investigate information about scene categories encoded by high versus low spatial frequencies. Behavioral studies have shown that when viewing a scene, low spatial frequency content of the image is processed prior to higher spatial frequencies [11]. This phenomenon is referred to as the coarse-to-fine hypothesis of visual perception [12]. A logical explanation for this finding is that low spatial frequencies provide the coarse description of the global attributes present in an image, therefore providing a sufficient amount of information for scene recognition. Characteristics such as edges, contours and other sharper details captured by high spatial frequencies are processed later in order to refine the initial coarse estimate of the image into a more detailed representation that includes local properties of the image. The coarse-to-fine hypothesis encapsulates the role of both low and high spatial frequency information in scene perception but strongly emphasizes the importance of global features captured primarily by low spatial frequencies [13].

The peripheral field bias observed in parahippocampal cortex lends neuroscientific support to the importance of low spatial frequencies for scene perception [14, 15]. According to this bias, PPA should show greater sensitivity to low spatial frequencies based on the fact that high spatial frequency tuning in early visual areas decreases at larger eccentricities [16]. On the other hand, recent observations show that high-spatial frequency contours that are preserved in line drawings of natural scenes [17] and, in particular, junctions of these contours [5, 6] play a critical role in scene categorization. Moreover, neuroimaging studies have shown that scene-selective brain regions are more strongly activated by high than low spatial frequencies [8, 18, 19]. Furthermore, Nasr et al. (2014) report that the PPA responds particularly to the presence of right angles [20], although this rectilinear edge selectivity has been found to be insufficient to explain category selectivity of the PPA [21].

Crucially, most of the aforementioned studies only compare overall activation levels, and do not address how much information about image *content* is conveyed by different spatial frequencies. One exception is Watson et al. (2016), who used a multivariate pattern analysis (MVPA) approach to analyze neural activation patterns elicited by high- and low-spatial frequency versions of indoor and natural images [10]. However, their approach was to contrast decoding of spatial frequency (high or low) with decoding of image content (indoor or natural), rather than comparing the amount of image content conveyed by each spatial frequency channel (i.e., decoding of image content for high *versus* low spatial frequency images).

To address the unresolved question “How much scene content is conveyed by different spatial frequencies in scene-selective visual cortex?” we presented participants with images of frequency-filtered scenes and compared how well scene categories could be decoded from fMRI activation patterns in each of the frequency conditions. Images from four different natural scene categories (beaches, forests, cities, and highways) were presented in three spatial frequency conditions (low spatial frequency filtered, high spatial frequency filtered, and unfiltered). We measured decoding accuracy in four brain regions involved in scene processing, the PPA (including its anatomical subdivisions—see below), the retrosplenial cortex (RSC), the occipital place area (OPA), and lateral occipital cortex (LOC).

Recent evidence suggests that rather than considering the PPA to be functionally homogenous, it should be thought of as anatomically differentiated. For instance, the PPA has been shown to overlap with several visual field maps [22] and disparate functional deficits have been observed in individuals with lesions in different locations of PPA [23]. These findings introduce the possibility that PPA is composed of multiple sub-regions that are functionally distinct, particularly along the anterior-posterior axis. Perhaps the most convincing work supporting this notion comes from Baldassano et al., (2013) who found differential functional connectivity along the anterior-posterior axis of PPA to distinct cortical networks [24]. They showed that posterior PPA (pPPA) has stronger connectivity with occipital areas including LOC and OPA, whereas anterior PPA (aPPA) has stronger connectivity with RSC and ventral prefrontal cortex. In the current study we first analyze our data in the PPA as a single ROI (along with OPA, RSC, and LOC), and then we subdivide PPA into aPPA and pPPA to test for differences along the anterior/posterior axis.

Finally, to illustrate the importance of scene content conveyed by these images, we contrast activation by frequency-filtered scenes with semantic-free filtered noise stimuli. Even though the filtered noise stimuli do not activate scene-selective brain regions to the extent that scenes do, they serve as an important control for explicit neural representations of spatial frequencies.

## Methods

### Participants

Data were analyzed from 10 subjects with normal or corrected-to-normal vision, between 18 and 26 years old (mean age: 20.8; 7 female). All participants were compensated financially for their time in the experiment. The experiment was approved by the Institutional Review Board of The Ohio State University, and all participants gave their written informed consent. Six other subjects participated but were disqualified because ROIs could not be delineated using their functional localizer data. An initial set of 10 subjects was collected using a TE = 22 ms, which was discovered after data collection. Low TEs can enhance anatomical signal but sometimes result in lower functional BOLD signal [25]. To assess functional data quality for these subjects, we examined data collected from the (independent) functional localizer task. Six of these subjects did not have sufficient signal quality to localize PPA and LOC from this standard functional localizer, so we did not analyze their data from the main experiment. We replaced them with 6 new subjects, who were naïve to the stimulus set, and collected their fMRI data with a TE = 30 ms. There was no difference in main experiment results as a function of TE for the included subjects.

### Stimuli

**Fig 1:**
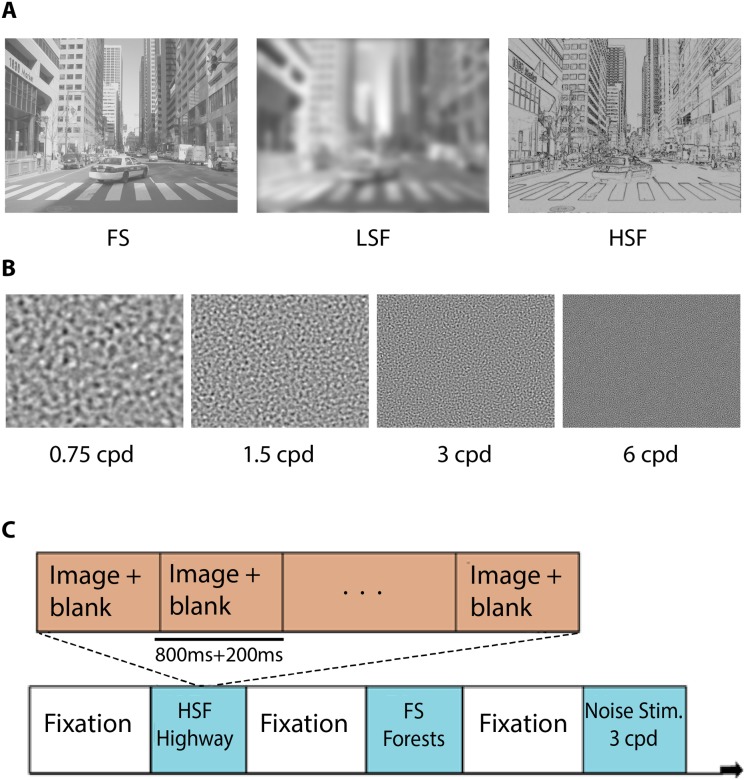
Examples of experiment stimuli and design. **(**A) Examples of a beach scene that was jointly contrast normalized across each spatial frequency condition: full spectrum (FS), low spatial frequencies (LSF) and high spatial frequencies (HSF). The four scene categories were beaches, highways, forests, and cities. (B) Examples of the noise stimuli at each of the four spatial frequencies included in the experiment. (C) Sample experiment sequence. Each of the eight runs consisted of 16 blocks of images (blue boxes; one per stimulus condition). Each block contained a sequence of eight different images of that condition.

In order to investigate the spatial-frequency specific response to naturalistic stimuli, we used grayscale photographs from four different natural scene categories: beaches, forests, cities and highways [26]. We generated three versions of each image (80 images per scene category): a full-spectrum (FS), unfiltered version; a low spatial frequency (LSF) version containing only spatial frequencies below 0.75 cycles per degree (cpd), corresponding to 17 cycles per image (cpi), low-pass filtered using a second-order Butterworth filter to avoid aliasing; and a high spatial frequency (HSF) version containing only spatial frequencies above 6 cpd, corresponding to 136 cpi, high-pass filtered with a second-order Butterworth filter. (Note that the common practice of deriving HSF images by subtracting a blurred version of the image from the original image results in ringing artifacts and spurious negative values in the HSF image. This is avoided by multiplying a properly tapered HSF filter with the Fourier amplitude spectrum of the image, while keeping the phase spectrum unchanged.) At no point during the experiment were any images repeated. Contrast polarity of the HSF images was chosen to depict dark edges on a light gray background. Following frequency filtering, the contrast of each set of images (meaning the FS, HSF, and LSF versions of a given scene image) was jointly normalized. To this end, the pixel values in each of the three versions of an image were adjusted to zero mean and unit standard deviation (z-scoring) and then linearly transformed jointly to the dynamic range [0,1]. As a result of this procedure, all three versions of an image had equal contrast and luminance (Fig 1A). Equating contrast and luminance between different filtered versions of the images is critical for eliciting comparable neural responses [19].

Additionally, we designed frequency-filtered noise stimuli in order to examine neural responses elicited by different spatial frequencies in the absence of semantically relevant information. These unstructured stimuli were constructed by band-pass filtering a uniform amplitude spectrum using a Butterworth filter at narrowly defined spatial frequency bands centered around 0.75, 1.5, 3 and 6cpd, corresponding to 17, 24, 68, and 136 cpi. This specially constructed amplitude was combined with a random phase spectrum and inverse-Fourier transformed into image space (Fig 1B). A total of 60 different filtered noise stimuli at each of the specified spatial frequencies were created and normalized to the same contrast and luminance.

### Experiment design

The experiment consisted of eight runs, each lasting approximately five minutes and thirty seconds. Every run contained 16 blocks, one per condition. The 16 different conditions were: 12 blocks of scene stimuli (one for each combination of the four scene categories and three spatial frequency conditions), and four blocks of the spatial frequency-filtered noise stimuli (one per frequency band). The order of the 16 blocks was randomized across runs and subjects. Each block lasted 8s and was followed by a 12s fixation period to allow the BOLD response to return to baseline.

Each block contained a sequence of 8 different images of the same stimulus condition (Fig 1C). Images were shown for 800 ms, followed by a 200 ms inter-stimulus interval consisting of a gray, mean-luminance background. Images were centered on the screen and covered 22.7 x 17 degrees visual angle. A fixation cross spanning approximately 0.7 degrees of visual angle was present at the center of the display throughout the entirety of each run. Participants were instructed before the start of each run to maintain fixation on the center cross and to simply view the scenes and stimuli displayed. Since we did not measure gaze location in the experiment, we relied on participants’ compliance with the fixation instructions. There were no explicit tasks required of the participants throughout the duration of the main experiment besides paying attention to the various scene and filtered noise stimuli.

### fMRI data acquisition

All scans were performed at the Center for Cognitive and Behavioral Brain Imaging (CCBBI) at The Ohio State University. Images were acquired using a 3-Tesla Siemens Total Imaging Matrix (TIM) Trio MRI scanner with a 32-channel head coil. For every subject, a high-resolution T1-weighted MPRAGE sequence was used to acquire structural images with 1x1x1mm voxels, two repetitions, TR = 1900 ms, and TE = 4.44 ms, lasting 6 minutes 50 seconds.

Functional data was obtained using a gradient echo, echo-planar sequence with 2x2x2 mm voxels, without gap, a partial coverage FOV consisting of 31 slices obliquely oriented to maximize coverage of occipital and occipito-temporal areas of cortex with a 67° flip angle for the main experiment and a 75° flip angle for the localizers. To assist in the co-registration of the partial coverage obliquely scanned functional data to the whole-brain anatomical data, a whole-brain gradient echo echo-planar sequence consisting of a single acquisition 25 seconds in length and oriented identically to the other functional runs was performed. For the main experiment a TR of 2000 ms was used, and for the face-place-object localizer a TR of 3000 ms was used. TE was 22 ms for four subjects and 30 ms for six subjects.

### Data analysis

FMRI data underwent standard preprocessing procedures using the fMRI analysis package AFNI [27]. Functional scans were motion corrected and co-registered to the anatomical MPRAGE in a single step in order to eliminate the need for multiple interpolations of the functional data. Data were spatially smoothed with a Gaussian kernel of 4 mm FWHM for all univariate analyses, including localizer scans, and 2 mm FWHM for all multivariate analyses. Functional data were normalized to percent signal change relative to fixation periods for each run.

Univariate analyses of the main experiment runs were performed on individual subject data using a standard general linear model (GLM). Incorporated into the full model were regressors for each of the four spatial frequency-filtered noise conditions (0.75, 1.5, 3 and 6 cpd), the FS, HSF, and LSF scenes (collapsed across scene categories), and fixation (no stimulus present). Nuisance variables for subject motion and low-frequency scanner drift were also included in the model. Regressors were convolved with a canonical hemodynamic response function (corresponding to the length of time each series of images was presented within each block), and a GLM analysis was performed using AFNI’s 3dDeconvolve, resulting in beta coefficients for each voxel.

For the multi-voxel pattern analysis (MVPA), we performed the following steps: Following motion correction, alignment to each subject’s anatomical volume and 2 mm spatial smoothing, fMRI data was subjected to a GLM analysis containing only nuisance regressors for subject motion as well as scanner drift. Residuals of this GLM analysis were extracted with a lag of 4 seconds to approximate the hemodynamic delay. For each participant, 512 functional volumes were extracted (8 runs x 16 blocks x 4 acquisitions per block). The four acquisitions obtained per block were subsequently averaged, resulting in 128 volumes per subject that were used for various decoding analyses.

A linear support vector machine classifier (SVM) [28] was trained to assign labels of either 1.) the scene category of the stimulus (4-way) or 2.) the spatial frequency of the stimulus (binary classification of high or low spatial frequency). For each participant, the classifier was trained on 7 of the 8 main experiment runs and tested on the left-out eighth run. This procedure was repeated eight times, leaving out each of the eight runs in turn (a leave-one-run-out, LORO, cross validation procedure). Due to the high dimensionality and low number of training exemplars, the training data are bound to be linearly separable. We therefore chose a low value for the C parameter (5·10^−6^) of the SVM, thereby favoring large margin decision boundaries, which lead to better out-of-sample generalization.

The scene category classification was performed separately for the FS, HSF, and LSF conditions. In each case, classification accuracy was calculated as the measure of interest; classification accuracy was compared to chance (25%) with planned, one-tailed t-tests. Comparison between conditions was performed using planned, two-tailed t-tests. Effect size was measured using Cohen’s d.

The spatial frequency classification was performed separately for scene stimuli (collapsing across scene categories), and for filtered noise stimuli (averaging across the 0.75 and 1.5 cpd conditions for LSF and the 3 and 6 cpd conditions for HSF). Classification accuracy was compared to chance (50%) using planned, one-tailed t-tests. Accuracy was compared between conditions with planned, 2-tailed t-tests. Effect size was measured using Cohen’s d.

### Regions of interest (ROI) localization

In addition to the main experiment, two runs (approximately 7 minutes and 30 seconds each) of an independent face-place-object localizer were performed. Participants were asked to perform a one-back task while viewing blocks of images of faces, scenes, objects and grid-scrambled objects. Images were sized 11.3 x 11.3 degrees (400 x 400 pixels) and centered on the screen; scene images were presented as FS and selected from a different database than the main task. Data underwent standard preprocessing, spatially smoothed using a 4 mm FWHM Gaussian kernel. Scene-selective areas (PPA, RSC and OPA) were defined on an individual subject basis using a linear contrast of neural activation elicited by scenes vs. faces and objects [29–32]. LOC was defined for each individual using a contrast of activation to objects vs. scrambled objects [33]. ROIs were delineated as contiguous clusters, using a threshold of *p* < 0.05, corrected for multiple comparisons at the cluster level. A stricter threshold was used where necessary to isolate clusters. PPA, OPA, and LOC were localized in both hemispheres for all participants. RSC was localized bilaterally in 9 participants and only on the left in one participant.

For the separation of PPA into anterior and posterior sub-regions, regions were spatially divided along the anterior-posterior axis such that the number of voxels was approximately equal in aPPA and pPPA.

### Behavioral experiment

We additionally performed a separate behavioral experiment to assess behavioral categorization performance for the same filtered and normalized scene images. Twenty undergraduate students from The Ohio State University (18–22 years old, mean 19.4; 11 female) participated in the experiment for partial course credit and gave written, informed consent. The experiment was approved by the Institutional Review Board of The Ohio State University. Participants were unfamiliar with the image set and had not participated in the fMRI experiment.

Images were shown on a CRT monitor with a refresh rate of 60 Hz, attached to a standard PC running Python using the Vision Egg package [34]. Participants were seated approximately 57 cm from the screen, and images subtended approximately 23° x 18° of visual angle.

In the experiment, participants were asked to categorize briefly presented images into one of four categories (beaches, forests, cities and highways) by pressing a key on a standard keyboard. Assignment of keys (‘s’,’d’,’j’,’k’) was randomized for each participant and learned during an initial practice phase. Images were immediately followed by a perceptual mask for 500 ms. During practice, the image-to-mask stimulus onset asynchrony (SOA) was fixed to 250 ms. Once participants achieved 90% accuracy, the SOA was staircased with a target performance of 70% correct using the QUEST staircasing algorithm [35]. Final SOAs ranged from 16.7 to 83.3 ms (M = 36.1 ms; SD = 13.1 ms). Two subjects’ data were excluded from the analysis because of low performance during staircasing (SOA > 150 ms).

Participants received acoustic feedback during practice and staircasing when they made an error. A set of 48 images was set aside for use in practice and staircasing, and only full-spectrum, unfiltered images were used.

During the test phase, a set of 240 new images was used; 20 images were presented in each combination of category (beaches, forests, cities, or highways) and experimental conditions (FS, HSF, or LSF). Assignment of images to the conditions was counter-balanced across participants, and the order of images in the experiment was randomized for each participant. SOA was fixed to the final value of the staircasing procedure for all conditions. No feedback was given. Response accuracy was calculated separately for the FS, HSF and LSF conditions, and accuracy was compared to chance (25%) using one-tailed t-tests and between conditions using 2-tailed t-tests.

## Results

### Decoding of scene category

Our primary question of interest was how much information regarding scene categories is conveyed by HSF and LSF scenes in the aforementioned regions. It has previously been shown that scene-selective areas of cortex (PPA, RSC and OPA) not only show greater relative levels of activation in response to places and buildings compared with faces and objects [29–32], but that the distributed patterns of activity elicited by different types of scenes contain *scene content* information, such that an image’s specific scene category (beach, highway, etc.) can be decoded from these regions [17, 26]. Accordingly, we performed multivariate decoding analyses in order to test whether spatial frequency content affects this category-specific information known to be present in these regions.

**Fig 2:**
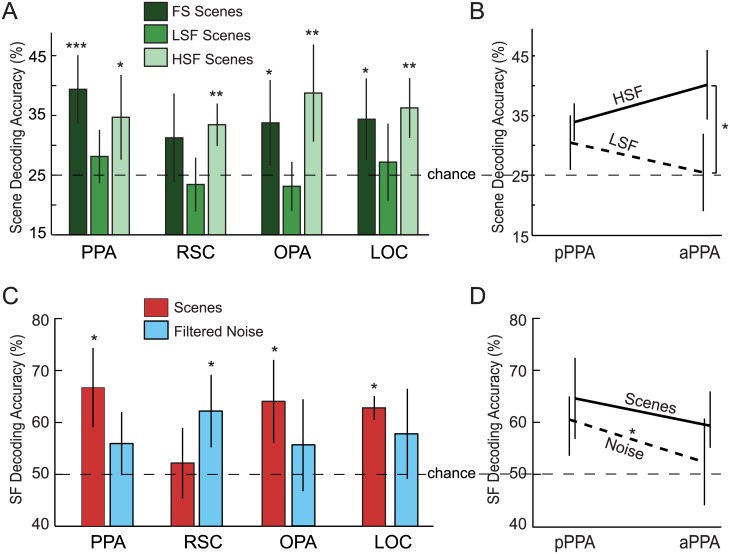
Multivariate decoding results. (A) Decoding of scene categories (4-way classification) is as accurate for HSF as for FS scenes. LSF scenes, on the other hand, cannot be decoded above chance. (B) Dividing the PPA along the A-P axis, reveals similar decoding accuracy for HSF and LSF scenes in posterior PPA, but significantly higher accuracy for HSF than LSF in anterior PPA. (C) Decoding of high versus low spatial frequencies from scene stimuli succeeded in PPA, OPA, and LOC but not the RSC. Decoding of spatial frequency from filtered noise stimuli succeeded only in RSC. (D) Decoding of spatial frequency was more accurate from the posterior than the anterior subdivision of the PPA. This difference was statistically significant for noise images but not for scenes. Error bars represent standard errors of the mean. *p < 0.05, **p < 0.01, ***p < 0.001; N = 10.

The decoder was trained and tested in a LORO cross-validation procedure to discriminate between four categories (beaches, forests, highways and cities). For unfiltered, full-spectrum images, decoding of scene categories was significantly above chance in all of the tested ROIs (PPA: *t*_*(9)*_ = 4.93, *p* < 0.001, *d* = 1.56; OPA: *t*_*(9)*_ = 2.39, *p* = 0.02, *d* = 0.76; LOC: *t*_*(9)*_ = 2.69, *p* = 0.01, *d* = 0.85), except for RSC, where it was marginal (*t*_*(9)*_ = 1.65, *p* = 0.07, *d* = 0.52). These results reproduce previous results showing neural representations of scene categories in these ROIs [26]. Of particular importance was testing how the spatial frequency content of the low- and high-pass filtered sets of scenes would impact the accuracy of the decoder in comparison to full-spectrum scenes. As illustrated in Fig 2A, scene categories were not decodable above chance (25%) when only low spatial frequency information was present in the image (PPA: *t*_*(9)*_ = 1.37, *p =* 0.10, *d* = 0.43; RSC: *t*_*(9)*_ = -0.68, *p* = 0.74, *d* = -0.22; OPA: *t*_*(9)*_ = -0.90, *p* = 0.80, *d* = -0.28; LOC: *t*_*(9)*_ = 0.66, *p* = 0.26, *d* = 0.21). On the other hand, it was possible to decode the categories of the HSF scenes above chance for all of the tested scene-selective ROIs (PPA: *t*_*(9)*_ = 2.68, *p =* 0.013, *d* = 0.85; RSC: *t*_*(9)*_ = 4.67, *p* < 0.001, *d* = 1.48; OPA: *t*_*(9)*_ = 3.31, *p* = 0.005, *d* = 1.05), as well as object-selective area LOC (*t*_*(9)*_ = 4.40, *p* < 0.001, *d* = 1.39). In fact, accuracy for HSF scenes was not significantly different from full-spectrum scene decoding accuracy in these ROIs (PPA: *t*_*(9)*_ = 1.20, *p* = 0.26, *d* = 0.38; RSC: *t*_*(9)*_ = 0.57, *p =* 0.58, *d* = 0.18; OPA: *t*_*(9)*_ = 1.15, *p =* 0.28, *d* = 0.36; LOC: *t*_*(9)*_ = 1.15, *p =* 0.28, *d* = 0.36). Planned comparisons between the LSF and HSF conditions showed that decoding accuracy was significantly higher for HSF scenes than LSF scenes in RSC *(t*_*(9)*_ = 2.95, *p =* 0.016, *d* = 0.93), OPA *(t*_*(9)*_ = 3.68, *p =* 0.005, *d* = 1.16) and LOC *(t*_*(9)*_ = 2.59, *p =* 0.029, *d* = 0.82). In the PPA, the effect went in the same direction, although not significantly *(t*_*(9)*_ = 1.86, *p =* 0.096, *d* = 0.59). See S1 Table for full results of individual participants.

Might the marginal effect in PPA reflect an anterior-posterior differentiation? HSF scenes were decodable above chance from both the posterior and anterior sub-divisions of PPA *(t*_*(9)*_ = 5.47, *p <* 0.001, *d* = 1.73 and *t*_*(9)*_ = 5.04, *p <* 0.001, *d* = 1.60, respectively), but, interestingly, LSF scene categories were only decodable in pPPA (*t*_*(9)*_ = 2.28, *p =* 0.024, *d* = 0.72), and not aPPA (*t*_*(9)*_ = 0.09, *p =* 0.47, *d* = 0.03). Moreover, decoding of scene categories was significantly more accurate for HSF than LSF scenes in the aPPA (*t*_*(9)*_ = 3.66, *p =* 0.005, *d =* 1.16) but not the pPPA (*t*_*(9)*_ = 1.06, *p =* 0.317, *d =* 0.335). This suggests that anterior and posterior PPA differ in the scene content carried by low versus high spatial frequencies.

### Decoding spatial frequency

As a secondary analysis, we also tested whether spatial frequencies themselves can be decoded from patterns of activity measured in these ROIs (Fig 2C; individual results in S2 Table). For this analysis, the same LORO cross-validation procedure was used for a binary discrimination between LSF and HSF, ignoring scene categories. The spatial frequency of the scene stimuli could be decoded above chance from PPA *(t*_*(9)*_ = 4.30, *p <* 0.001, *d* = 1.36), OPA *(t*_*(9)*_ = 3.45, *p =* 0.004, *d* = 1.09) and LOC *(t*_*(9)*_ = 11.05, *p* < 0.001, *d* = 3.49) but not from RSC *(t*_*(9)*_ = 0.63, *p =* 0.27, *d* = 0.20). Decoding of spatial frequency for scenes succeeded for both subdivisions of PPA (pPPA: *t*_*(9)*_ = 3.70, *p =* 0.002, *d* = 1.17, aPPA: *t*_(9)_ = 3.22, *p =* 0.005, *d* = 1.02, Fig 2D).

Does this mean that the spatial frequencies of the stimuli are directly accessible in these brain regions, independent of image content and structure? To answer this question we also attempted to decode spatial frequency from the filtered noise stimuli. For this analysis we grouped together the two lowest spatial frequencies (0.75 and 1.5 cpd) and the two highest spatial frequencies (3 and 6 cpd). Spatial frequency of the filtered noise stimuli could be decoded significantly above chance from RSC (*t*_*(9)*_ = 3.42; *p* = 0.004; *d* = 1.08) and PPA (*t*_*(9)*_ = 1.91, *p* = 0.044, *d* = 0.61), marginally from LOC (*t*_*(9)*_ = 1.77, *p* = 0.06, *d* = 0.56), but not from OPA (*t*_*(9)*_ = 1.25, *p* = 0.12, *d* = 0.39; Fig 2C). We also found an interesting dissociation between the anterior and posterior subdivisions of PPA. We could decode the spatial frequency of the filtered noise stimuli significantly above chance from pPPA *(t*_*(9)*_ = 3.84; *p =* 0.002; *d* = 1.22) but not aPPA *(t*_*(9)*_ = 0.59; *p =* 0.29; *d* = 0.19; Fig 2D), and the difference in decoding accuracy between pPPA and aPPA was statistically significant (*t*_*(9)*_ = 2.29; *p =* 0.048; *d* = 0.72). This result implies that spatial frequencies are explicitly represented in posterior PPA but are accessible in anterior PPA only when they are associated with a scene image.

### Univariate analyses

**Fig 3:**
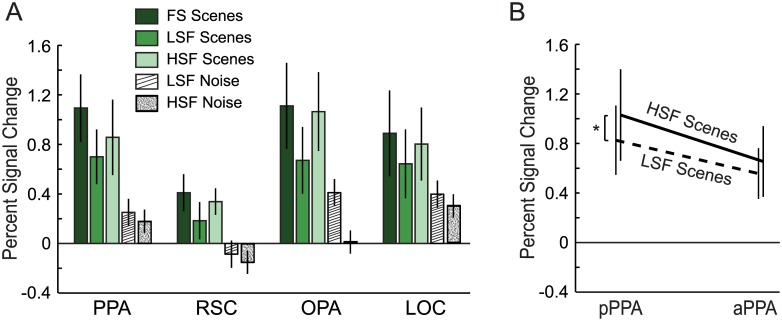
Percent signal change in visual ROIs. (A) Univariate activation of high-level visual ROIs by spatial frequency-filtered scenes and noise stimuli. (B) HSF scenes activate pPPA more strongly than LSF scenes. This difference does not persist in aPPA. Error bars are standard errors of the mean. *p < 0.05.

We conducted a univariate analysis testing whether the different scene-selective ROIs (PPA, RSC, and OPA, along with LOC) exhibited an overall preference for high versus low spatial frequency images (Fig 3A). HSF scenes tended to elicit a higher response in these ROIs compared to LSF scenes. This effect was significant in OPA *(t*_*(9)*_ = 3.08, *p* = 0.013, *d* = 0.97) and marginal in PPA, RSC, and LOC *(t*_*(9)*_ = 1.89, *p =* 0.091, *d* = 0.60; *t*_*(9)*_ = 1.99, *p* = 0.077, *d* = 0.63; and *t*_*(9)*_ = 2.10, *p =* 0.065, *d* = 0.66; respectively). We found no significant difference in mean activation elicited by HSF vs. LSF scenes in the anterior PPA (*t*_*(9)*_ *=* 1.02, *p =* 0.335, *d* = 0.32), but pPPA responded significantly more to HSF than LSF scenes (*t*_*(9)*_ *=* 2.58, *p =* 0.030, *d* = 0.82; Fig 3B; individual results in S3 Table).

High vs. low spatial frequency comparisons within each ROI for the filtered noise stimuli (where the two lowest spatial frequencies and two highest spatial frequencies were collapsed) yielded no significant differences (PPA: *t*_*(9)*_ = 0.82, *p =* 0.43, *d* = 0.26; RSC: *t*_*(9)*_ *=* 0.53, *p =* 0.61, *d* = 0.16; OPA: *t*_*(9)*_ *=* 1.76, *p =* 0.11, *d* = 0.56; LOC: *t*_*(9)*_ = 0.89, *p =* 0.40, *d* = 0.28; pPPA: *t*_*(9)*_ = 0.92, *p =* 0.38, *d* = 0.29; aPPA: *t*_*(9)*_ = 0.63, *p =* 0.55, *d* = 0.20). These null findings are likely due to the fact that the filtered noise stimuli did not activate scene-selective areas very much.

### Behavioral experiment

In the behavioral experiment, participants categorized the scenes in all three conditions clearly above chance (FS: 78.2%, *t*_*(17)*_ = 22.4, *p* < 0.001; HSF: 69.0%, *t*_*(17)*_ = 15.6, *p* < 0.001; LSF: 41.3%, *t*_*(17)*_ = 11.8, *p* < 0.001; for full results see S4 Table). Performance was significantly lower in the two modified conditions than in the full-spectrum condition (HSF versus FS: *t*_*(17)*_ = 6.2, *p* < 0.001; LSF versus FS: *t*_*(17)*_ = 23.1, *p* < 0.001)., Behavioral performance was also significantly less accurate in the LSF condition than in the HSF condition (*t*_*(17)*_ = 11.4, *p* < 0.001).

## Discussion

Here we took a novel approach to the question of how visual cortex constructs representations of scene content, specifically asking whether activity patterns in scene-selective cortex are more sensitive to scene content conveyed by high or low spatial frequency information. Previous studies have compared overall activation levels to high versus low spatial frequency images [8, 18, 19], or have compared decoding of low-level information (spatial frequency) to decoding of high-level information (scene category) [10]. By contrast, the current study tests whether decoding of high-level scene content is preferentially driven by certain spatial frequencies. This approach is similar to the recent proposal of investigating “feature diagnosticity” for scene perception [36].

We suggest that such an approach to exploring the tuning properties of high-level visual areas is better suited for uncovering how image content and low-level image features relate than juxtaposing these two aspects of visual information. Pitching measurable low-level visual features of the images against high-level image content such as scene categories and object identities (e.g., [7, 9, 10]) implies that in principle the two are separate. We instead advocate for a model of perception in which image content is tightly intertwined with low-level features. That is, image content cannot exist independently of low-level features, even though it can be conveyed by a variety of features. Thus, we should seek to understand which features in what combination can convey how much information about image content. In doing so here, we have found that the content of scenes in these areas of human visual cortex are predominantly represented through high spatial frequency channels. Additionally, we show the nature in which spatial frequency is processed in the aforementioned areas, and perhaps most interestingly, an important functional distinction of pPPA and aPPA in how spatial frequency information is processed.

Our primary finding is that decoding of scene category information was more accurate for high spatial frequency compared to low spatial frequency images. This finding runs counter to a long held mantra regarding the importance of low spatial frequencies in scene perception, notably that low spatial frequencies contain more information about scene gist [11,37] as well as global attributes of scenes such as openness and expansiveness [3, 4, 38]. This also raises the question of how well our HSF and LSF images conveyed scene category at a behavioral/perceptual level. We directly tested this question in an additional behavioral experiment, in which participants saw briefly-presented backward-masked HSF and LSF images and reported the scene category of each image. While categorization was possible from both versions of the images, categorization using HSF images was significantly more accurate than categorization using LSF images. We do not argue against the sentiment that low spatial frequency information plays a role in scene perception, but perhaps it is not as fundamental in these occipito-temporal areas of cortex—and for analogous perceptual tasks—as the high spatial frequency components contained in a scene. This idea is consistent with recent neuroimaging studies showing that scene-selective brain regions are more strongly activated by high than low spatial frequencies [8, 10, 18, 19]. Critically, here we go further by showing that representations of scene *content* are also more strongly conveyed by high than low spatial frequencies. This idea has been hinted at in less direct ways, e.g., by several recent behavioral and neuroimaging studies highlighting the importance of edge features that define the geometric structure of scenes [17, 20, 39] and demonstrating that information contained in edge-only line drawings of scenes carries category-specific information that matches human behavior [5] as well as the neural representation of scene categories in PPA [6].

One explanation for this HSF advantage is that the clearer representation of objects and surfaces as well as their spatial relations in HSF scenes is advantageous for conveying scene content. However, attention may also play a role, since attentional engagement may be higher for HSF scenes that contain clearly distinguishable object information than for LSF scenes with blurred, apparently washed-out object information. We cannot discriminate between these two interpretations based on the current empirical results, and both factors may underlie the perceptual advantage of HSF scenes to some extent.

The importance of HSF information in the PPA also appears to be at odds with findings of over-representation of the peripheral visual field in the PPA [14, 15, 22]. Low spatial frequency information tends to be more dominant in peripheral areas, which would predict the opposite pattern to our findings. High spatial frequencies are also posed to be filtered out by early-level neurons due to hierarchy and eccentricity [40], which raises the question of where the PPA’s HSF information originates. One possible resolution would be to call upon feedback signals from higher up in the visual processing hierarchy, which would deliver detailed information about shape and structure to the PPA. High spatial frequency information might also reach the PPA via other visual pathways bypassing early visual cortex [24]. Another possibility is that the PPA’s reported preference for high eccentricities is rooted in its defining preference for places over objects; in other words, that more eccentric stimuli provide more place-like structure and may be processed as more scene-like by the PPA.

The second main contribution of this study is the dissociation between posterior and anterior portions of the PPA. Previous studies have found differential functional connectivity along the anterior-posterior axis of PPA, suggesting that they form distinct cortical networks [24]. Recent work also suggests that the posterior region of PPA plays a significant role in processing low-level visual properties (e.g. spatial frequencies, contrast, color and luminance) and the anterior region of PPA is relatively less important regarding these image properties [20, 24]. Our results generally support this view: spatial frequency information could be decoded from both scenes and semantic-free noise stimuli in pPPA, but only from the scene images in aPPA. This distinction is important because scenes inherently contain structure and contextual meaning beyond low-level visual features. By testing the noise stimuli designed to have equivalent luminance and contrast along with no semantically relevant scene information, we showed that pPPA carries explicit spatial frequency representations and that aPPA does not. The finding of more spatial frequency information in pPPA is compatible with results showing a retinotopic map in two areas in the posterior parahippocampal cortex [22]. Because we did not conduct retinotopic mapping here, we cannot be certain that those areas correspond to our pPPA, yet retinotopic organization of these areas could underlie a decodable representation of spatial frequencies, since the periphery would preferentially respond to LSF and foveal regions to HSF.

We also found that pPPA responded significantly more to HSF scenes than LSF scenes in the univariate analysis, consistent with Rajimehr et al. (2011) [8]. Interestingly, the other area that showed a significant univariate difference for HSF greater than LSF was the OPA; Baldassano et al.’s (2013) functional connectivity analysis showed that pPPA has stronger connectivity with occipital areas including LOC and OPA, whereas aPPA has stronger connectivity with RSC and ventral prefrontal cortex [24].

In our experiments, scene content could be decoded from HSF scenes in all visual areas tested (PPA, pPPA, aPPA, RSC, OPA, and LOC), but the scene categories of LSF scenes could only be decoded above chance from neural activation patterns of the pPPA. Several theories state that information flows from coarse to fine [12, 13] or simple to complex [32, 41] when moving from posterior scene areas (e.g. OPA) to more anterior PPA and then RSC. Our results do not entirely fit within this framework given the lack of LSF scene decoding in OPA. This might be explained by a different division of labor, with OPA performing a different function in scene perception, such as navigation [42].

Our findings open up other interesting questions for future study. For example, here we examined only low SF filtered (≤ 0.75 cpd) versus high SF filtered (≥ 6 cpd) scene images—it would be interesting if the spatial frequencies that best convey scene content followed a posterior-to-anterior gradient in PPA if more frequency channels were tested. Note that HSF information of the kind used here (>6 cpd) is only available at or near the fovea. Accessing the full image content beyond the initial fixation will therefore require eye movements, which should be addressed in future work. It will also be important to extend this experimental design to testing the contributions of other types of low-level information, such as color, texture, or motion, to representing stimulus content. In combining the jigsaw pieces from such tests, a clearer image will emerge of how the human visual system transforms collections of low-level features so that high-level concepts become directly accessible.

## Supporting information

S1 Table Individual data for decoding scene category. (XLSX)

S2 Table Individual data for decoding spatial frequency. (XLSX)

S3 Table Individual data for univariate brain activity. (XLSX)

S4 Table Individual results for the behavioral experiment. (XLSX)
